# Microbial vitamin production mediates dietary effects on diabetic risk

**DOI:** 10.1080/19490976.2022.2154550

**Published:** 2022-12-06

**Authors:** Daoming Wang, Van T. Pham, Robert E. Steinert, Alexandra Zhernakova, Jingyuan Fu

**Affiliations:** a Department of Genetics, University of Groningen, University Medical Center Groningen, Groningen 9713AV, the Netherlands; b Department of Pediatrics, University of Groningen, University Medical Center Groningen, Groningen 9713AV, the Netherlands; c Global R&D Center Human Nutrition and Care (HNC), DSM Nutritional Products Ltd, Basel, Switzerland; d Department of Surgery, Division of Visceral and Transplantation Surgery, University Hospital Zurich, Zurich, Switzerland

**Keywords:** Human gut microbiome, vitamin metabolism, cardiometabolic health, diabetes, exposures, fruit intake

## Abstract

Adequate levels of essential vitamins are important for the prevention of diabetes. While the main efforts to address this are currently focused on the intake of vitamin supplements, improving and maintaining intrinsic vitamin production capacity, which is determined by gut microbes, has received insufficient attention. In this study, we systematically investigated the relationship between gut microbial vitamin production and factors related to diabetes and cardiometabolic health in a deeply phenotyped cohort, Lifelines-DEEP (N = 1,135). We found that blood glucose–related factors, lipids, circulating inflammation, and fecal short-chain fatty acids are associated with gut microbial vitamin production. Use of laxatives and metformin are associated with increased levels of vitamin B1/B6 biosynthesis pathways. We further reveal a mediatory role for microbial vitamin B1/B2 production on the influence of fruit intake on diabetes risk. This study provides preliminary evidence for microbiome-targeted vitamin metabolism interventions to promote health.

## Introduction

Diabetes care remains a major public health and socioeconomic burden, and this burden is set to rise, with over 500 million people worldwide predicted to be living with type 2 diabetes (T2D) by 2030.^1^ Vitamins are essential micronutrients involved in normal physiology and the cardiometabolic health of humans,^2^ and abnormal vitamin levels are often observed in patients with diabetes, including vitamin D deficiency^3^ and abnormal urinary loss of vitamin C.^4^ Moreover, higher levels of folate and vitamin B12 in red blood cells during early pregnancy have been associated with a higher risk of gestational diabetes.^5^ Long-term metformin use to treat T2D can induce vitamin B12 deficiency, which leads to hematologic abnormalities, progressive axonal demyelination, and peripheral neuropathy.^6^ Maintaining a healthy vitamin supply in the body is thus a crucial element of diabetes prevention and treatment.

As most vitamin species, especially members of the vitamin B, K, and E families, cannot be synthesized by the human body, dietary intake is a direct way to obtain vitamins. However, many factors influence the bioavailability of diet-derived vitamins. The content of temperature-sensitive vitamins can decrease during the process of food storage, while the solubility of vitamins and cooking processes also impact bioaccessibility and absorption of dietary vitamins in the digestive tract.^7,8^ Finally, the high knowledge threshold for understanding dietary nutrients and socioeconomic factors have also limited the prevalence of a balanced daily diet in the general population.

Beside the external intake of vitamins through diet and supplements, another important source of vitamins is intrinsic vitamin production by the gut microbiome. Vitamin biosynthesis genes and pathways have been discovered in many common gut bacterial species, e.g. *Bacteroides fragilis* and *Bacteroides thetaiotaomicron*.^9^ A populational scale metagenomic investigation revealed that there is an abundance of vitamin production pathways in many gut bacteria^10^ and showed that a balanced gut microbiome is crucial for intrinsic vitamin production. For example, a deficiency of vitamin B12 related to *Heliobacter pylori* infection was shown to be caused by altered gut microbiome structure.^11^ Bacterial production of vitamins can also be involved in disease development.^12^ For instance, gut microbial pathways related to riboflavin and thiamine production are associated with the activity status of Crohn’s disease and decrease during disease exacerbations,^13^ and enrichment in the bacterial vitamin K2 production pathway has been observed in diabetes.^14^

Although the role of the gut microbiome in diabetic risk has been explored in several studies,^15,16^ few have focused on bacterial vitamin production and its role in mediating the effects of lifestyle and medication in diabetes. Therefore, we performed a focused analysis on gut microbial vitamin production pathways and investigated their association with diabetic risk and many other clinical and lifestyle factors in the deeply phenotyped Dutch cohort Lifelines-DEEP (N = 1,135).

## Materials and methods

### Lifelines-DEEP cohort

Lifelines-DEEP (LLD) is a sub-cohort of Lifelines, a large population-based prospective cohort that enrolled 167,729 participants from the northern Netherlands to explore the risk factors behind complex diseases. In LLD, 1,539 Lifelines individuals were included and multi-layers of omics data were collected. For the current study, we examined high-quality metagenomic sequencing data, 78 dietary factors, 5 smoking factors, and 44 drug usage factors that were available for 1,135 individuals (474 male and 661 female). The average age of LLD participants was 45.04 years old (18‒81, SE = 0.40). The average BMI was 25.26 (16.67–48.56, SE = 0.12).

### Metagenomic sequencing and quality control

Microbial DNA was isolated from fecal samples of LLD participants and sequenced as previously described.^17,18^ We removed host genome‒contaminated reads and low-quality reads from the raw metagenomic sequencing data using KneadData (version 0.7.4), Bowtie2 (version 2.3.4.3),^19^ and Trimmomatic (version 0.39).^20^ In brief, the data-cleaning procedure includes two main steps: (1) filtering out of human genome‒contaminated reads by aligning raw reads to the human reference genome (GRCh37/hg19) and (2) removing of adapter sequences and low-quality reads using Trimmomatic with default settings (SLIDINGWINDOW:4:20 MINLEN:50).

### Taxonomic and pathway abundance

We generated the taxonomic relative abundance and pathway abundance profile for LLD from the cleaned metagenomic reads using MetaPhlAn2^21^ and HUMAnN.^22^ We further extracted all gut microbial pathways involved in vitamin biosynthesis based on the MetaCyc classification “Vitamin Biosynthesis”, which yielded abundance levels for 28 vitamin biosynthesis pathways. We also generated grouped abundance values by grouping the abundances of different pathways from the same vitamin type. Both the individual and grouped abundances of vitamin biosynthesis pathways were used for downstream analysis. The Shannon index was calculated using the function *diversity()* of the R package *vegan* (version 2.6–2).

### Quantification and statistical analysis

All statistical tests were performed using R (version 4.0.1). Details of statistical tests are also provided in the Results section and figure legends.

### Association analysis

Spearman’s rank correlation was used to identify the associations between species abundance and pathway abundance, without correcting for any covariates. This resulted in 4,648 tests, with the false discovery rate controlled using the Benjamini-Hochberg method. Differences in microbial vitamin production pathways between males and females were analyzed using the Wilcoxon rank-sum test, followed by false discovery control of multiple-testing P values using the Benjamini-Hochberg method. Associations between age and relative abundance of microbial vitamin production pathways were conducted using Spearman’s rank correlation, and sex-related heterogeneity was checked using Cochran’s Q test.

Before performing association analysis between microbial pathway abundance and other host-related factors, we standardized all continuous variables to follow a standard normal distribution (*N~(0, 1)*) using empirical normal quantile transformation. Associations between microbial vitamin pathways and host-related factors were assessed using a linear model (*Model 1*):

Pathway abundance ~ age + sex + factor

We then confirmed the robustness of the significant associations identified by *Model 1* (FDR < 0.05) using MaAsLin2^23^ with two extra models: one considering age and sex as fixed effect covariates (*Model 2*) and another considering all health factors or exposures as fixed effect covariates (*Model 3*).

### Mediation analysis

The regulatory relationships between the frequency of fruit intake, vitamin B1/B2 pathways, and glucose-related factors were inferred by bidirectional mediation analysis using the R package *mediation* (version 4.5.0). In detail, we used fruit intake frequency as a predictor or treatment factor, vitamin B1/B2 pathways as mediators, and glucose-related factors as outcomes in direction 1. We then swapped mediators and outcomes in direction 2. In each direction, the mediation analysis contained three main steps: (1) assessment of the effect of the treatment factor on the mediator using a linear model, (2) assessment of the effect of the mediator on the outcome while controlling for the treatment factor using a linear model, and (3) assessment of the indirect effect of the treatment factor on the outcome through the mediator using the function *mediate()* from R package *mediation* with the parameter *boot = T* and the fitted models generated in step 1 and step 2 as inputs (Supplementary material 1).

## Results

### Overview of microbial vitamin pathways

**Figure 1.**
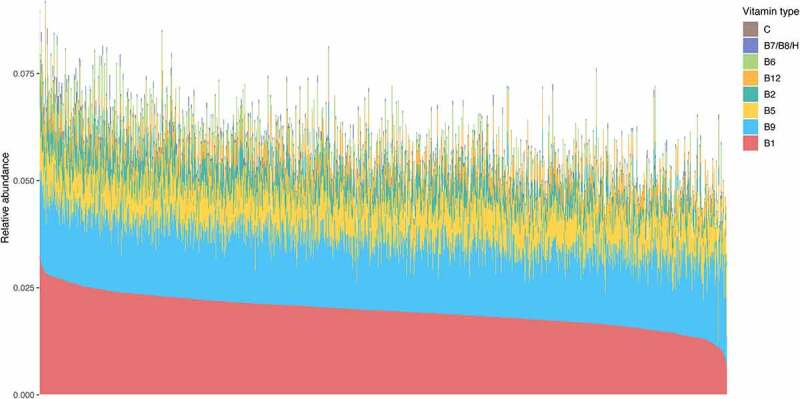
**Relative abundance of grouped bacterial vitamin pathways in all samples**. Samples were sorted by the relative abundance of B1.

Circulating vitamin levels have important implications for human health, with vitamin levels determined by diverse factors including diet, the gut microbiome, and human genetics.^24^ As many vitamin species in the human body can only be sourced from diet or the gut microbiome, we hypothesized that inter-individual variation in bacterial vitamin production capacity in the gut microbiome may have an impact on human health. To assess this, we extracted the abundance levels of 28 vitamin biosynthesis pathways from previously reported metagenomics data from the LLD cohort.^18^ These pathways involved the production of vitamin Bs (B1, B2, B5, B6, B7/B8/H, B9, and B12) and one vitamin C pathway (Table S1). Summary statistics of the individual pathways and the eight grouped pathways can be found in Tables S2 and S3. On average, these 28 vitamin biosynthesis pathways collectively accounted for 5.8% of the total microbial functional profile, with a range of 2.8% to 9.2% (Figure 1). The top two vitamin production pathways were related to B1 (thiamine) and B9 (folate), which accounted for an average of 1.97% and 1.65% of the total microbial functional profile, respectively (Figure 1, Table S3).

To identify microbial species that may be involved in vitamin production, we computed associations between abundance levels of vitamin biosynthesis pathways and 166 common species that were present in over 10% of the samples. This detected 1,333 positive associations at FDR < 0.05 level (Table S4). We then confirmed some of the vitamin-producing species. For instance, PWY-6167, a flavin (B2) biosynthesis II pathway, was previously identified in Archaea,^25^ and we observed a very strong association between PWY-6167 abundance and the abundance of the Archaea species *Methanobrevibacter smithii* (R = 0.95, P = 0). We further identified many other potential vitamin-production species, including *Escherichia coli* for the production of thiamin diphosphate (B1) and salvage of pyridoxine (B6), *Eubacterium rectale* for cobalamine (B12) salvage, and *Collinsela aerofaciens* for folate (B9) biosynthesis. Figure S1 shows the top 12 associations, which are all related to vitamin B production. For vitamin C, *Odoribacter splanchnicus* may be the major contributing species (R = 0.54, P = 2 × 10^−85^), followed by *E. coli* (R = 0.26, P = 1 × 10^−17^), and *Clostridium hathewayi* (R = 0.21, P = 3 × 10^−12^). Additionally, we also linked the alpha-diversity of the gut microbiome to the relative abundance of grouped vitamin pathways, and vitamin B2 showed the strongest positive associations with the Shannon index of the gut microbiome, while vitamin B12 showed the strongest negative association (Spearman’s rank correlation, P < .05; Figure S2).

### Sex differences and age-related changes in bacterial vitamin pathways

The abundance levels of total bacterial vitamin biosynthesis pathways of B6 and B2 were higher in females than males in our cohort, whereas B12 biosynthesis was enriched in males (Table S5). At the individual pathway level, 6 out of 28 pathways showed a significant difference between the sexes (Wilcoxon rank-sum test, FDR < 0.05; Table S6). We also observed differences in vitamin biosynthesis. For example, while total B1 pathways showed no difference between males and females (Wilcoxon rank-sum test, P = .3; Figure S3A) at the individual pathway level, males had a higher vitamin B1 biosynthesis pathway from pyrithiamine and oxythiamine (PWY-7357), whereas females could produce more B1 from pyridoxine (PWY-7282) (Figure S3B and C; Table S5 and S6).

**Figure 2.**
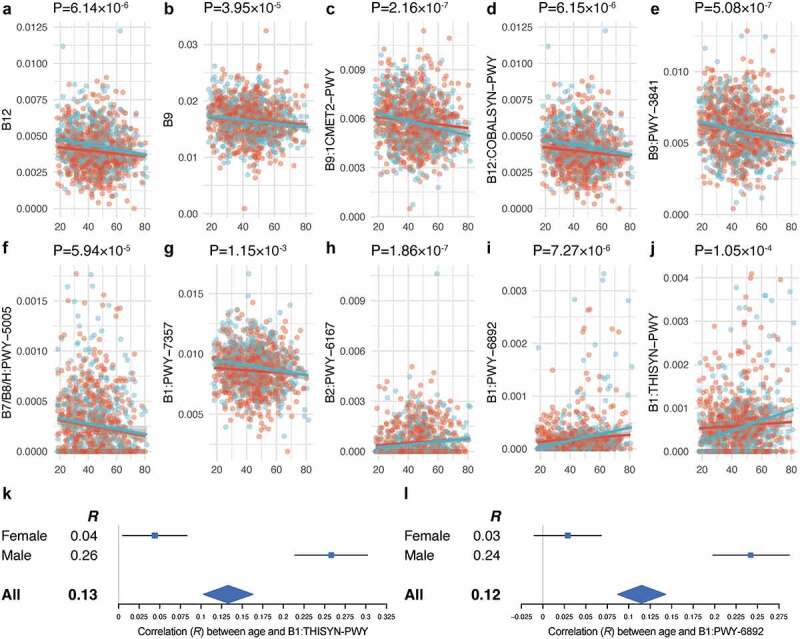
**The trend of microbial vitamin production pathways with age. a–j**. Significant associations between age and bacterial vitamin pathways (Spearman’s rank correlation, FDR < 0.05). Red and blue circles represent females and males, respectively. **k–l**. Forest plots show a sex-dependent increase in the abundance of B1: THISYN-PWY (k) and B1: PWY-6892 (l) with increasing age.

Total vitamin B9 and B12 biosynthesis pathways, as well as five individual pathways, were negatively correlated with age (Spearman’s rank correlation, FDR < 0.05; Tables S7 and S8; Figure 2a-g). The strongest age-associated decrease was for the formation of the formyl and methyl derivatives of vitamin B9 (1CMET2-PWY, R = −0.15, P = 2.16 × 10^−7^). To our surprise, the relative abundance of three pathways increased with age: riboflavin biosynthesis from GTP and D-ribulose 5-phosphate (PWY-6167, R = 0.15, P = 1.86 × 10^−7^) and thiamine diphosphate (B1) biosynthesis from pyrimidine and thiazole moieties (THISYN-PWY) and from its precursor biosynthesis pathway of thiazole moieties (PWY-6892). Interestingly, the age-related increasing trends of PWY-6892 and the THISYN-PWY are sex-dependent and only seen in males (Cochran’s Q test, P < .05; Figure 2k and il; Table S8).

### Gut microbial vitamin production correlates with diabetes-related factors

**Figure 3.**
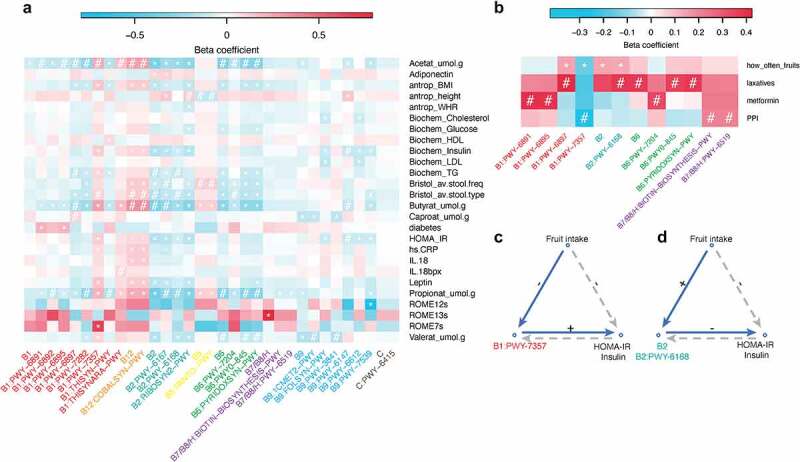
**Associations of gut microbial vitamin production with health-related and exposure factors. a**. Significant associations between health-related factors and bacterial vitamin pathways identified by linear regression. **b**. Significant associations between exposure factors and bacterial vitamin pathways identified by linear regression. In the cells of the heat maps a • indicates that the corresponding association is significant in *Model 1* (FDR < 0.05) but not confirmed by *Model 2* (FDR > 0.05) or *Model 3* (P > .05), a * represents that the corresponding association is significant in *Model 1* (FDR < 0.05) and confirmed by *Model 2* (FDR < 0.05), and a # represents that the corresponding association is significant in *Model 1* (FDR < 0.05) and confirmed by *Model 3* (P < .05). The beta coefficients used for the heatmap coloring are based on the results of *Model 1*. **c–d**. Microbial vitamin B1 and B2 pathways mediated the effect of fruit-intake frequency on HOMA-IR and insulin (P_mediation_ < 0.05). * FDR < 0.05. **c**. Vitamin B1 pathway as the mediator. **d**. Vitamin B2 pathways as the mediators. + indicates a positive effect. – indicates a negative effect. Solid blue lines show the regulatory direction of fruit-eating–vitamin pathway–glucose factors (direction 1). Gray dashed lines indicate the regulatory direction of fruit-eating–glucose factors–vitamin pathways (direction 2).

To investigate the relationship of intrinsic vitamin biosynthesis from the gut microbiome with diabetic risk and general health, we assessed associations with T2D and its related parameters, including plasma levels of HbA1c, glucose, and insulin and homeostatic model assessment for insulin resistance (HOMA-IR). As diabetes patients also show other metabolic and inflammation-related syndromes, we also included blood lipid levels, plasma levels of cytokines and adipokines, Bristol stool type, and fecal levels of short-chain fatty acids (SCFAs) that are important microbiome-derived components implicated in diabetes. In total, we included 58 health-related factors in our analysis using a linear model that included age and sex as covariates. This identified 199 significant associations for 26 health-related factors (*Model 1*, FDR < 0.05, Table S9): 54 positive and 145 negative associations (Figure 3a, Table S9). Of the 199 significant associations identified by *Model 1*, 152 and 50 associations were also identified using MaAsLin2 in *Model 2* (Figure 3a; Table S9; FDR < 0.05) and *Model 3* (Table S10; P < .05), respectively.

Bacterial biosynthesis of B2 and B9 were negatively associated with diabetes and its related parameters, including HOMA-IR, insulin level, glucose level, and diabetes. *Bacteroides uniformis*, the species showing a strong association with flavin biosynthesis III in our data (vitamin B2: PWY-6168, Table S4), was previously found to reduce serum glucose level in mice by oral administration.^26^ Interestingly, we found the grouped B1 and B12 pathways were positively associated with HOMA-IR.

To exclude the effects of medication, we further included drugs used as covariates. Even after this accounting for drug usage, the grouped B12 pathway, thiamine diphosphate biosynthesis III (B1:THISYNARA-PWY), and thiamine phosphate formation (B1:PWY-7357) were still positively associated with HOMA-IR and insulin level (P < .05; Table S11). In our previous study,^27^ PWY-7357 was associated with blood protein levels of the paraoxonases family 3 (PON3) and plasminogen activator inhibitor (PAI), and the levels of both PON3 and PAI are mainly determined by gut microbiome factors and are associated with increased glucose level, insulin level, and diabetes risk.^27,28^

Vitamin B has also been implicated in the regulation of lipid metabolism^29,30^ and obesity.^31^ We detected 12 associations of vitamin Bs with blood lipid levels, with 11 of the 12 being negative associations with the plasma level of low-density lipoprotein cholesterol and triglyceride levels (Table S9). A recent study reported the depletion of biotin (vitamin B7/B8/H) producers in the gut of severely obese patients.^31^ In line with this, we also found that the gut microbial production potential of biotin was negatively associated with BMI in our general population cohort (Figure 3a; *Model 2*, FDR < 0.05). In addition to metabolism, bacterial vitamin biosynthesis pathways were found to be linked to inflammation and cytokine levels. In our data, the bacterial flavin biosynthesis II pathway (PWY-6167) is negatively associated with the plasma level of high-sensitive C-reactive protein (P = 2.98x10^–5^), an inflammatory marker. This pathway also showed a negative association with leptin (P = 9.36x10^–5^). These findings collectively indicate a close relationship between gut microbial vitamin production and dyslipidemia and chronic inflammation.

SCFAs have been shown to protect against cardiometabolic diseases and have been implicated in diabetes care.^32^ We found that fecal levels of five SCFAs (propionate, butyrate, acetate, valerate, and caproate) were associated with 30 bacterial vitamin pathway variables (FDR < 0.05, Table S9). The Vitamin B5 and B12 pathways were positively associated with SCFAs, whereas vitamins B2, B6, B7, B9, and C were negatively associated with SCFAs.

### Medication usage and fruit intake influence gut microbial vitamin production

Our data has revealed microbial vitamin production pathways that are significantly associated with direct and indirect factors in diabetes. Since the modulation of intrinsic vitamin production capacity can have beneficial implications for metabolic health, we aimed to identify environmental exposures that influence gut microbial vitamin production. Diet, medication, and other lifestyle factors (e.g. smoking) have previously been associated with alteration in microbial composition in the LLD cohort.^18^ Out of the 127 lifestyle factors available for LLD (78 dietary factors, 44 drug usages, and 5 smoking-related factors), four were associated with bacterial vitamin pathways at FDR < 0.05: fruit intake and use of laxatives, metformin, or proton pump inhibitors (PPIs) (Figure 3b, Table S12), with 10 and 11 out of the 15 associations with exposures also identified by *Model 2* (Figure 3b; Table S12; FDR < 0.05) and *Model 3* (Table S13; P < .05), respectively. It has been widely reported that metformin can induce B12 deficiency.^6,33^ However, we did not detect a significant association for the bacterial B12 biosynthesis pathway. Instead, metformin increased the abundance of bacterial pathways for B1 and B6 biosynthesis, with the top pathway being synthesis of the thiazole moiety of thiamin (PWY-6891) (Beta = 0.40, P = 5.50 × 10^−7^; Table S12). Laxative use showed the highest number of associations with bacterial vitamin pathways, and the most significant association was with PWY-6897 (B1) (Beta = 0.42, P = 4.49 × 10^−6^; Table S12). Interestingly, our data show diverse effects of fruit-intake frequency on the biosynthesis of vitamin B1. Fruit-intake frequency was positively associated with thiamine phosphate formation from pyrithiamine and oxythiamine (PWY-6897) (Beta = 0.14, P = 7.74 × 10^−5^; Figure 3b; Table S12) but negatively associated with the thiamine diphosphate salvage pathway (PWY-7357) (Beta = −0.15, P = 2.23 × 10^−5^; Figure 3b; Table S12). These two pathways even seemed to be carried out by different species, as the species most strongly associated with PWY-6897 was *Bacteroidales bacterium* ph8 (R = 0.46, P = 1.1 × 10^−59^), whereas the species most strongly associated with PWY-7357 was *Ruminococcus bromii* (R = 0.39, P = 6.2 × 10^−43^; Table S4). The positive effect of fruit intake on vitamin B2 production is consistent with our previous finding that the presence of plant-derived protein in the diet increases the level of the vitamin B2 pathway PWY-6168.^34^

### Fruit intake-related decreases in diabetes risk are associated with bacterial vitamin B1 and B2 production

To provide clues about gut microbiome–targeted dietary interventions for health improvement, we investigated if bacterial vitamin production capacity mediated the effects of the dietary factor on disease risk. As we had observed significant associations between fruit-intake frequency and gut microbial vitamin B1 and B2 pathways, and gut microbial vitamin B1 and B2 pathways are associated with glucose-related health factors (insulin level, glucose level, HOMA-IR, and diabetes), we performed bidirectional mediation analysis between fruit-intake frequency, gut microbial vitamin B1 and B2 pathways, and glucose-related health factors. This analysis revealed that fruit-intake frequency significantly influences the bidirectional regulation between glucose-related indices (HOMA-IR and insulin level) and microbial vitamin production capacities (vitamin B1: PWY-7357, vitamin B2: PWY-6168, and vitamin B2 group) (P_mediation_ < 0.05; Figure 3c-d; Table S14), which suggest that fruit-derived proteins and components may decrease diabetes risk through regulation of gut bacterial production of vitamins B1 and B2.

## Discussion

Although the benefits of vitamin supplements on general health have been widely evaluated, the importance of intrinsic vitamin production by gut microbes in modulating the influence of lifestyle and medication factors on diabetes risk and general cardiometabolic health has received scant attention. This is the first comprehensive evaluation of the relationship between gut microbial vitamin production, diabetes, and cardiometabolic health–related and exposure factors. Our results reveal sex differences and age-related trends in microbial vitamin production and identify associations between gut microbial vitamin pathways and health-related factors as well as medical and dietary factors that influence gut microbial vitamin production, suggesting the value of microbiome-targeted interventions to adjust intrinsic vitamin production and improve wellbeing.

The age- and sex-related gut microbial characterizations have been well described before in different populations, including this cohort,^35^ but no study had specifically zoomed in on the gut microbial vitamin production pathways. We find that gut microbial capacity to produce vitamin B12 is higher in males, despite serum vitamin B12 levels being higher in females in previous studies in healthy populations.^36,37^ Thus, the sex difference in circulating vitamin B12 level may be also caused by other factors, e.g. vitamin B12 absorption capacity and interactions between sex and genetic factors.^36^ The abundance of gut microbial vitamin B12 production pathways also showed an age-related decline, suggesting that age-related microbiome alteration also contributes to decreased vitamin B12 levels in the older population.^38^

Gut microbiome composition and vitamin production capacity can regulate the production and consumption of glucose, which can further influence the risk of diabetes.^39^ Our results highlight the complex role of microbial vitamin pathways. In combination with our previous findings, the microbial vitamin B1 pathway PWY-7357 may increase the risk of diabetes by increasing the level of PAI in the blood. Interestingly, the activity of PAI-1 in plasma was found to be reduced in subjects with high fruit and vegetable consumption,^40^ and microbiome factors are the main contributors to PAI level in the blood.^27^ Vitamin B2 has inflammation-modulating properties,^41^ and our study shows that microbial vitamin B2 production was positively associated with anti-inflammatory adiponectin and negatively correlated with inflammatory marker C-reactive protein and pro-inflammatory leptin. Adiponectin and leptin are important hormones and cytokines (adipokines) generated by adipocytes and are closely involved in the obesity-induced inflammation process and insulin resistance.^42^ Supplementation of vitamin B2 was found to decrease serum levels of inflammatory markers in patients with Crohn’s disease^43^ and to relieve inflammation caused by oxidative stress in diabetic mice.^44^ A previous study had also revealed that changes in gut microbiome composition impact the expression of adipocytes in mice,^45^ our study suggests that vitamin B2 could be a molecular mediator between gut bacteria and adipocytes and highlights the role of gut microbial vitamin B2 production in reducing obesity-induced inflammation and insulin resistance.

The mediating role we found for microbial vitamin B1 and B2 production capacity between fruit consumption and diabetes risk highlights the potential of gut microbiome–targeted dietary interventions to promote health. However, the underlying mechanisms are unknown. Fruits are rich in dietary fiber, antioxidants, and other beneficial nutrients that have a complicated influence on the intestinal environment and microbial community and further modify the gut microbial composition. One possible hypothesis is that the dietary fiber enriched in fruit can promote intestinal motility, aid the maintenance of intestinal mucus layer structure,^46^ and serve as a substrate promoting the growth of beneficial bacteria, including vitamin-producing bacteria. It has been widely observed that fiber intake improves the intestinal microenvironment and increases gut microbial diversity,^18,47,48^ while the vitamin B2 biosynthesis pathway is prevalent and conserved in many gut bacteria^1^°. Indeed, we observed that increased gut microbiome diversity was positively associated with the abundance of the vitamin B2 biosynthetic pathway. Our findings suggest that fruit consumption can be used as a dietary intervention that targets multiple gut bacterial vitamin pathways to counterbalance factors that promote diabetes risk.

Both SCFAs and vitamins are beneficial metabolites for humans, and there is a close relationship between microbial vitamin production and fecal SCFA levels. SCFAs and vitamins are crucial participants in host energy metabolism and act in the metabolic interface of host–microbe interaction. SCFAs are mainly derived from non-digestible carbohydrates; they are produced by commensal bacteria in the gut and can be further converted to glucose. Glucose-derived pyruvates can be converted to acetyl-coenzyme A, which is the main input of the tricarboxylic acid (TCA) cycle. Most B group vitamins are necessary participants in a series of biochemical reactions that are part of the TCA cycle and directly contribute to energy metabolism.^49^ Vitamin B12, for example, can accelerate the metabolic rate of gut bacteria and further increase the level of SCFAs in the intestine, especially butyrate and propionate,^50^ and we also observed positive associations between vitamin B12 biosynthesis pathways and SCFAs including acetate, butyrate, and propionate. As gut bacteria are essential players in the production of SCFAs and vitamins, the coupling of SCFAs and vitamins in the energy metabolism process highlights the importance of microbiome-targeting interventions for the maintenance and improvement of host energy production.

We evaluated the impacts of dietary factors, drug usage, and smoking habits on the relative abundance of microbial vitamin production pathways, with use of PPIs, laxatives, and metformin found to correlate with vitamin pathways. Although metformin has been reported to have many positive impacts on human health,^51^ risk of vitamin B12 deficiency has been associated with metformin treatment in patients with T2D.^52^ We did not observe a significant association between metformin use and the microbial vitamin B12 pathway. Instead, metformin use was positively associated with two bacterial B1 pathways. Previous studies revealed that metformin is an inhibitor of the human thiamine transporter (THTR-2), which can influence the intestinal absorption of vitamin B1.^53^ Thus, improvement of vitamin B1 absorption may be helpful and efficient in the prevention of vitamin B1 deficiency in diabetes patients who use metformin. Usage of PPIs or laxatives was previously reported to strongly correlate with gut microbiome composition and diversity^18,54^ and should be further evaluated as a potential microbiome-targeted drug for vitamin metabolism intervention.

We acknowledge several limitations of this study. First, although we comprehensively investigated the relationships between gut microbial vitamin production pathways in a general population-based cohort, only Dutch participants were included. Considering the distinct gut microbiome compositions documented across the different populations with diverse nationalities, genetic backgrounds, and environments, replication in other populations is needed. Second, we performed the association study in a cross-sectional dataset and therefore cannot provide insights into causality between gut microbial vitamin production pathways and host-related factors. Thus, further confirmation of our results through longitudinal study and wet-lab validation is required. Finally, the gut microbial vitamin production pathway is not the only intrinsic factor that influences vitamin levels in human plasma and feces. Host genetic factors may also directly or indirectly impact the absorption of a given vitamin, and this should be considered in future studies, together with measurements of vitamin levels in human samples.

## Conclusion

Our results indicate that the levels of the gut microbial production pathways of vitamin B2 and B6 are higher in females than in males, but B12 production is higher in males than females. We also found gut microbial production of vitamin B9 and B12 to be decreased in middle-aged and older participants. Further, gut microbial vitamin production was related to factors directly and indirectly related to diabetes, including blood glucose, insulin, HOMA-IR, lipids, circulating inflammation, and fecal SCFAs. Use of laxatives and metformin were associated with the levels of vitamin B1 and B6 biosynthesis in our cohort. Finally, our mediation analysis found that microbial vitamin B1 and B2 production mediates the impact of fruit intake on diabetes risk, which highlights the importance of knowledge about the gut microbiome in guiding a healthy diet.

## Supplementary Material

Supplemental Material

## Data Availability

Raw metagenomic sequencing data of Lifelines-DEEP is publicly available at the European Genome‒Phenome Archive via accession numbers EGAS00001001704.
