# Supplementary material for: Microbial vitamin production mediates dietary effects on diabetic risk
Source: Gut Microbes. 2022 Dec 6;14(1):2154550. doi: 10.1080/19490976.2022.2154550 (PMC9733697; doi:10.1080/19490976.2022.2154550)
Supplement: Supplemental Material [file KGMI_A_2154550_SM9136.zip › Supplementary material 1.docx]

**Supplementary material 1**

# R code for mediation analysis

my_lm_mediation<-function(input.inv, input.med, input.dv, covDf){

input.df<-data.frame(input.inv,input.dv,input.med,covDf)

input.df.rmna<-na.omit(input.df)

input.df.rmna<-apply(input.df.rmna, 2, qtrans) %>% as.data.frame

if(length(table(input.df.rmna$input.inv))>1 &

length(table(input.df.rmna$input.dv))>1 &

length(table(input.df.rmna$input.med))>1

){

if(length(table(input.df.rmna$input.inv)) < 3 &

sort(table(input.df.rmna$input.inv),decreasing = T)[2] <= 10){

res_list <- list(N = nrow(input.df.rmna),

inv_med.beta=NA, inv_med.p=NA,

med_dv.beta=NA, med_dv.p=NA,

inv_dv.beta=NA, inv_dv.p=NA,

ACME.beta=NA, ACME.p=NA,

ADE.beta=NA, AED.p = NA,

Total.effect=NA, Total.effet.p=NA,

Prop.mediated = NA, Prop.mediated.p = NA)

}else{

fit.totaleffect=lm(input.dv~.,input.df.rmna[,-3])

fit.totaleffect.res<-summary(fit.totaleffect)

fit.mediator=lm(input.med~., input.df.rmna[,-2]) # input.inv

fit.mediator.res<-summary(fit.mediator)

fit.dv=lm(input.dv~.,input.df.rmna)

fit.dv.res<-summary(fit.dv)

results <- mediate(fit.mediator, fit.dv, covariates = colnames(covDf),

treat='input.inv', mediator='input.med', boot=T)

res<-summary(results)

res_list <- list(N = nrow(input.df.rmna),

inv_med.beta=fit.mediator.res$coefficients[2,1], inv_med.p=fit.mediator.res$coefficients[2,4],

med_dv.beta=fit.dv.res$coefficients[3,1], med_dv.p=fit.dv.res$coefficients[3,4],

inv_dv.beta=fit.totaleffect.res$coefficients[2,1], inv_dv.p=fit.totaleffect.res$coefficients[2,4],

ACME.beta=res$d0, ACME.p=res$d0.p,

ADE.beta=res$z0, AED.p = res$z0.p,

Total.effect=res$tau.coef, Total.effet.p=res$tau.p,

Prop.mediated = res$n0, Prop.mediated.p = res$n0.p)

}

}else{

res_list <- list(N = nrow(input.df.rmna),

inv_med.beta=NA, inv_med.p=NA,

med_dv.beta=NA, med_dv.p=NA,

inv_dv.beta=NA, inv_dv.p=NA,

ACME.beta=NA, ACME.p=NA,

ADE.beta=NA, AED.p = NA,

Total.effect=NA, Total.effet.p=NA,

Prop.mediated = NA, Prop.mediated.p = NA)

}

return(res_list)

}

## Bidirectional mediation analysis for the linear model

lm_bimediation<-function(inVec, indvDf, dvDf1, dvDf2, covDf, covar ){

if(is.na(inVec[4])){

covar<-covar

}else{

covar<-c(covar, colnames(covDf)[grep(inVec[4],colnames(covDf))])

}

indv<-indvDf[,match(inVec[1], colnames(indvDf))]

dv1<-dvDf1[,match(inVec[2], colnames(dvDf1))]

dv2<-dvDf2[,match(inVec[3], colnames(dvDf2))]

dir1_res <- my_lm_mediation(indv, dv1, dv2, covDf[,covar])

dir2_res <- my_lm_mediation(indv, dv2, dv1, covDf[,covar])

names(dir1_res)<-paste("dir1.",names(dir1_res),sep = "")

names(dir2_res)<-paste("dir2.",names(dir2_res),sep = "")

MediationDirection<-"none"

if(!is.na(dir1_res$dir1.Prop.mediated.p) & !is.na(dir2_res$dir2.Prop.mediated.p)){

if( dir1_res$dir1.Prop.mediated.p<0.05 & dir2_res$dir2.Prop.mediated.p<0.05){MediationDirection <- "both"}

if( dir1_res$dir1.Prop.mediated.p<0.05 & dir2_res$dir2.Prop.mediated.p>0.05){MediationDirection <- "indv_dv1_dv2"}

if( dir1_res$dir1.Prop.mediated.p>0.05 & dir2_res$dir2.Prop.mediated.p<0.05){MediationDirection <- "indv_dv2_dv1"}

}

bires<-list(indv=inVec[1], dv1=inVec[2], dv2=inVec[3],MediationDirection = MediationDirection)

res<-c(bires,dir1_res,dir2_res)

return(res)

}
