# Supplementary figures and images for: Microbial vitamin production mediates dietary effects on diabetic risk
Source: Gut Microbes. 2022 Dec 6;14(1):2154550. doi: 10.1080/19490976.2022.2154550 (PMC9733697; doi:10.1080/19490976.2022.2154550)

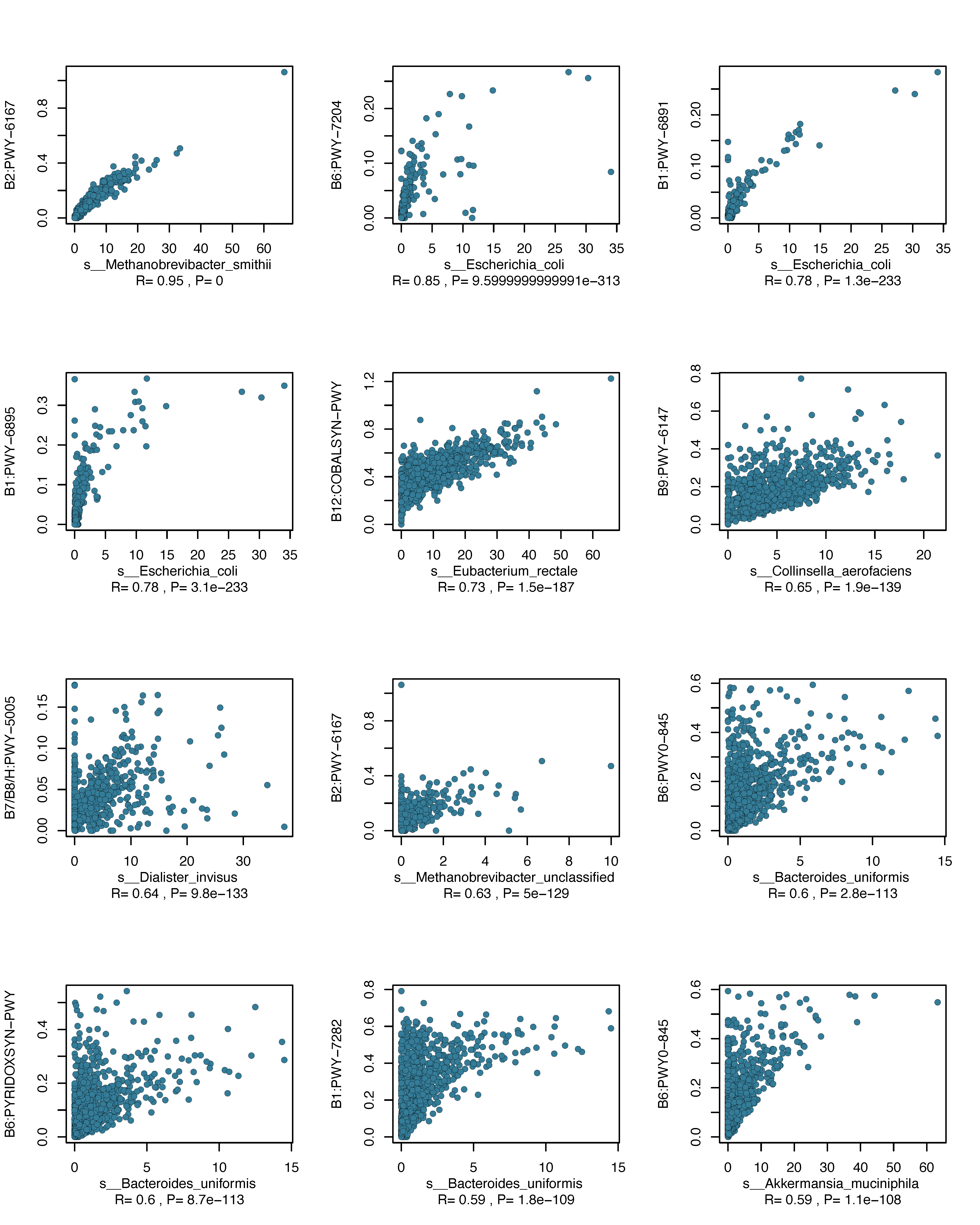

Supplement: Supplemental Material [file KGMI_A_2154550_SM9136.zip › Figure_S1.tiff]

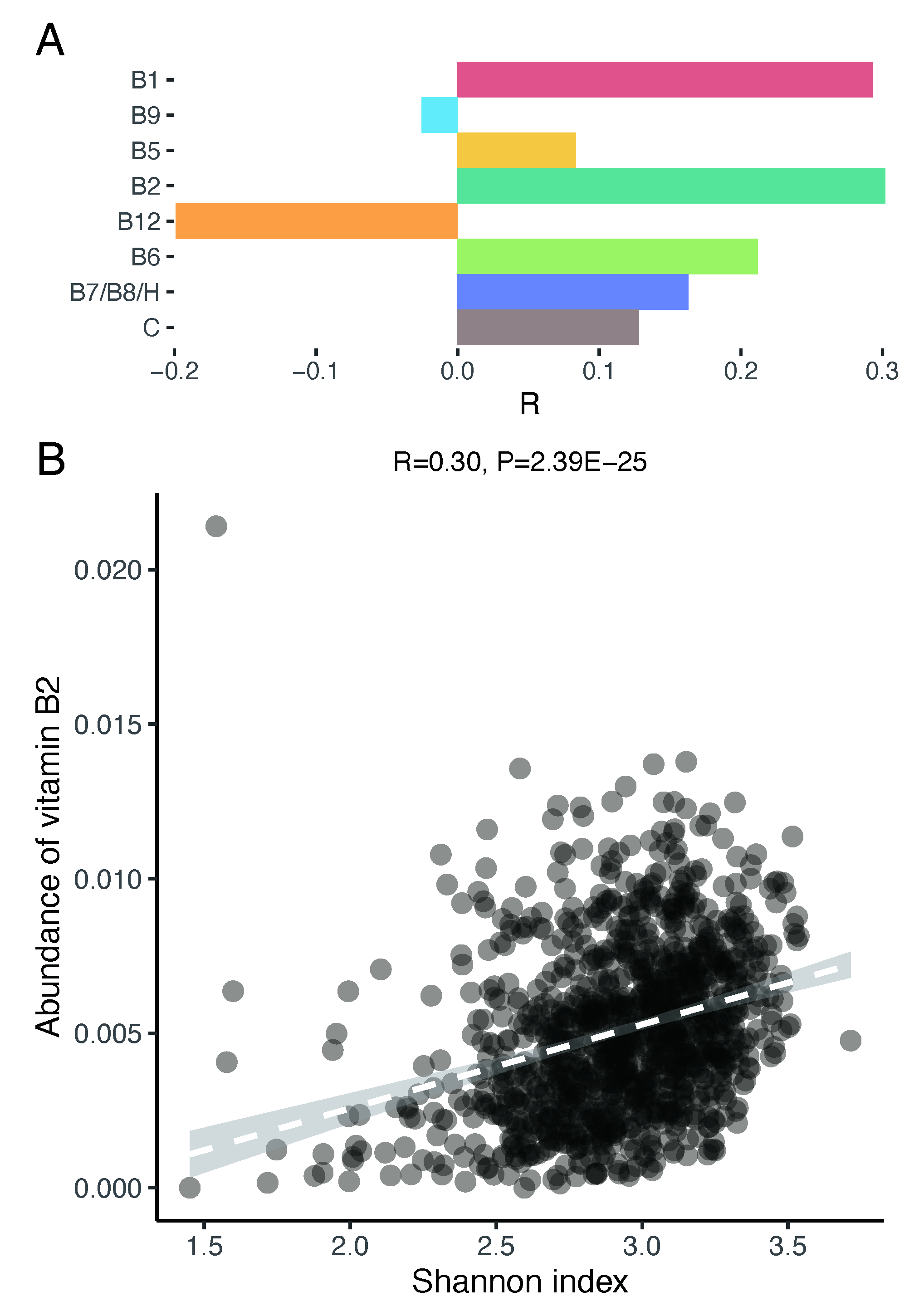

Supplement: Supplemental Material [file KGMI_A_2154550_SM9136.zip › Figure_S2.tiff]

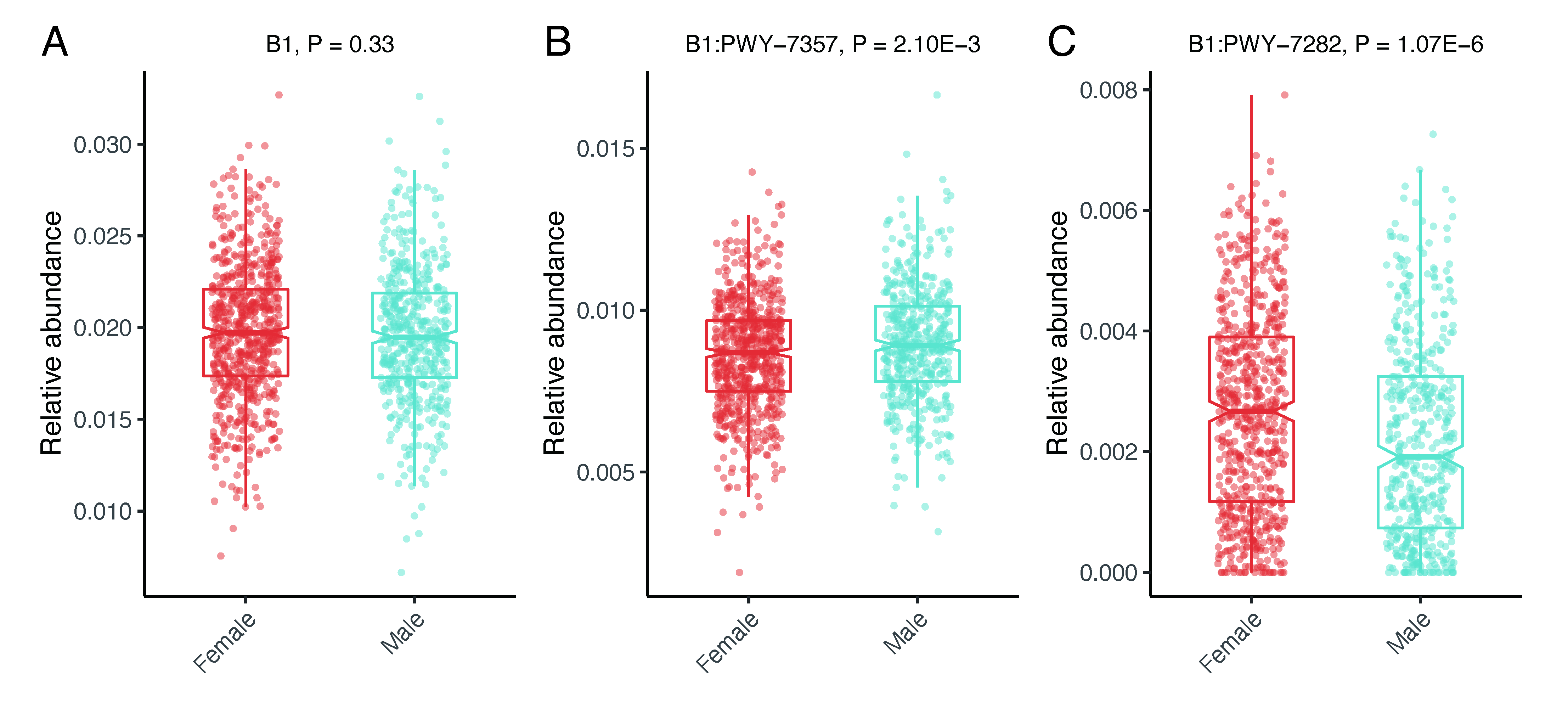

Supplement: Supplemental Material [file KGMI_A_2154550_SM9136.zip › Figure_S3.tiff]
